# Physics and Chemistry of Two-Dimensional Triangulene-Based Lattices

**DOI:** 10.1021/acs.accounts.4c00557

**Published:** 2024-12-10

**Authors:** Hongde Yu, Yu Jing, Thomas Heine

**Affiliations:** † Faculty of Chemistry and Food Chemistry, TU Dresden, Bergstrasse 66c, 01069 Dresden, Germany; ‡ Jiangsu Co-Innovation Centre of Efficient Processing and Utilization of Forest Resources, College of Chemical Engineering, Nanjing Forestry University, Nanjing 210037, China; § Helmholtz-Zentrum Dresden-Rossendorf, Centrum for Advanced Systems Understanding, CASUS, Untermarkt 20, 02826 Görlitz, Germany; ∥ Department of Chemistry, Yonsei University and IBS center for nanomedicine, Seodaemun-gu, Seoul 120-749, Republic of Korea

## Abstract

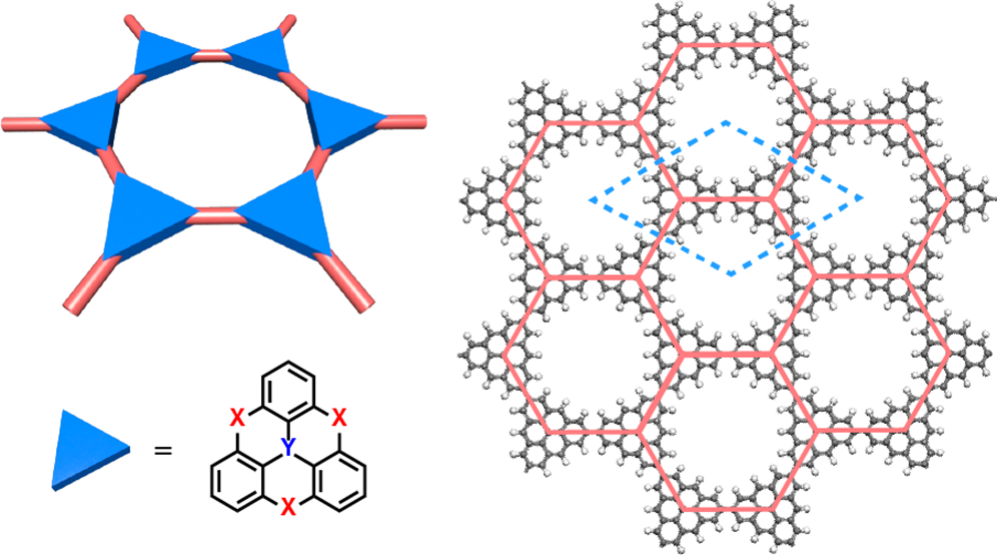

Triangulene (TRI) and its heterotriangulene (HT) derivatives are planar, triangle-shaped molecules that, via suitable coupling reactions, can form extended organic two-dimensional (2D) crystal (O2DC) structures. While TRI is a diradical, HTs are either closed-shell molecules or monoradicals which can be stabilized in their cationic form.

Triangulene-based O2DCs have a characteristic honeycomb-kagome lattice. This structure gives rise to four characteristic electronic bands: two of them form Dirac points, while the other two are flat and sandwich the Dirac bands. Functionalization and heteroatoms are suitable means to engineer this band structure. Heteroatoms like boron and nitrogen shift the Fermi level upward and downward, respectively, while bridging groups and functionalized triangulene edges can introduce a dispersion to the flat bands.

The stable backbone architecture makes 2D HT-polymers ideal for photoelectrochemical applications: (i) bridge functionalization can tune the band gap and maximize absorption, (ii) the choice of the center atom (B or N) controls the band occupation and shifts the Fermi level with respect to vacuum, allowing in some cases for overpotential-free photon-driven surface reactions, and (iii) the large surface area allows for a high flux of educts and products.

The spin polarization in TRI and in open-shell HTs is maintained when linking them to dimers or extended frameworks with direct coupling or more elaborate bridging groups (acetylene, diacetylene, and phenyl). The dimers have a high spin-polarization energy and some of them are strongly magnetically coupled, resulting in stable high-spin or broken-symmetry (BS) low-spin systems. As O2DCs, some systems become antiferromagnetic Mott insulators with large band gaps, while others show Stoner ferromagnetism, maintaining the characteristic honeycomb-kagome bands but shifting the opposite spin-polarized bands to different energies. For O2DCs based on aza- and boratriangulene (monoradicals as building blocks), the Fermi level is shifted to a spin-polarized Dirac point, and the systems have a Curie temperature of about 250 K. For half-filled (all-carbon) systems, the Ovchinnikov rule or, equivalently, Lieb’s theorem, is sufficient to predict the magnetic ordering of the systems, while the non-half-filled systems (i.e., those with heteroatoms) obey the more involved Goodenough–Kanamori rule to interpret the magnetism on the grounds of fundamental electronic interactions.

There remain challenges in experiment and in theory to advance the field of triangulene-based O2DCs: Coupling reactions beyond surface chemistry have to be developed to allow for highly ordered, extended crystals. Multilayer structures, which are unexplored to date, will be inevitable in alternative synthesis approaches. The predictive power of density-functional theory (DFT) within state-of-the-art functionals is limited for the description of magnetic couplings in these systems due to the apparent multireference character and the large spatial extension of the spin centers.

## Key References

- SpringerM. A.; LiuT.-J.; KucA.; HeineT.Topological two-dimensional polymers. Chem. Soc. Rev.2020, 49, 2007–201932206766 10.1039/c9cs00893d.^1^ This tutorial review discusses the fundamental structure–property relations of two-dimensional (2D) lattices and their realization as framework materials based on molecular building blocks stitched together with covalent bonds maintaining 2D conjugation.
- JingY.; ZhouZ.; GengW.; ZhuX.; HeineT.2D Honeycomb-Kagome Polymer Tandem as Effective Metal-Free Photocatalysts for Water Splitting. Adv. Mater.2021, 33, e200864533942398 10.1002/adma.202008645PMC11468641.^2^ Light absorbance and electrode reactions in the hydrogen and oxygen evolution reactions for photocatalytic water splitting are calculated for diamagnetic HT-based 2D polymers. A tandem electrode (z-scheme) composed of an aza- and a bora-HT is proposed for overpotential-free water splitting.
- YuH.; HeineT.Magnetic Coupling Control in Triangulene Dimers. J. Am. Chem. Soc.2023, 145, 19303–1931137610306 10.1021/jacs.3c05178PMC10485925.^3^ We show that typical coupling reactions between TRI and HTs maintain the spin polarization of the monomeric building blocks. In some cases, we observe a very large magnetic coupling.
- YuH.; HeineT.Prediction of metal-free Stoner and Mott-Hubbard magnetism in triangulene-based two-dimensional polymers. Sci. Adv.2024, 10, eadq795439356753 10.1126/sciadv.adq7954PMC12697527.^4^ We predicted Mott–Hubbard antiferromagnetism in TRI-based and Stoner ferromagnetism in aza- and bora-heterotriangulene organic two-dimensional (2D) crystals (O2DCs).

## 1 Introduction

**Figure 1:**
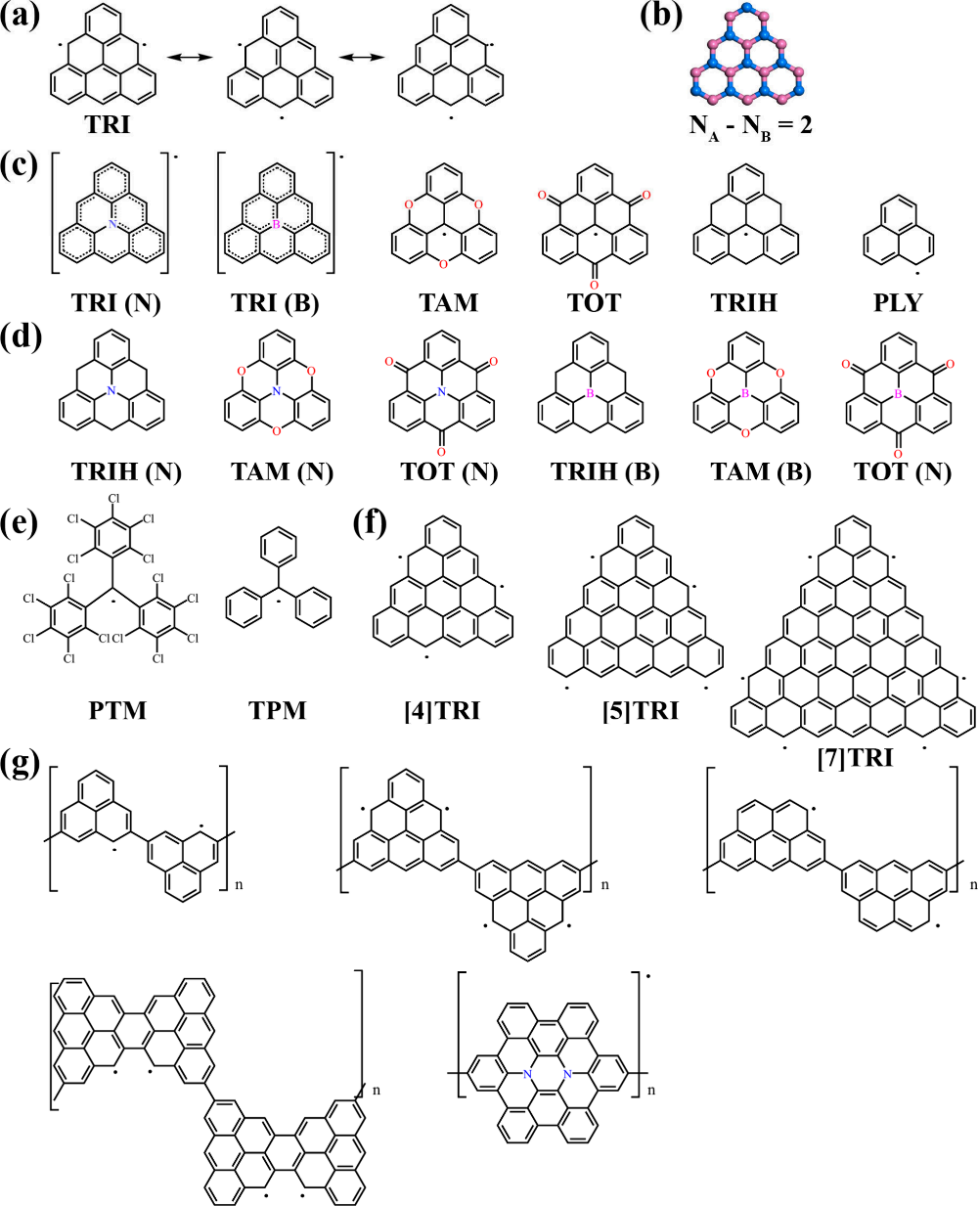
(a) Resonance structures of triangulene (TRI). (b) A and B sublattices of TRI shown in blue and red, respectively. Chemical structures of (c) TRI-based monoradicals, (d) closed-shell heterotriangulenes (HTs), and (e) nonplanar ring-opening derivatives (TPM and PTM). (f) TRIs with different sizes. (g) 1D spin chains made of nanographenes.

**Figure 2:**
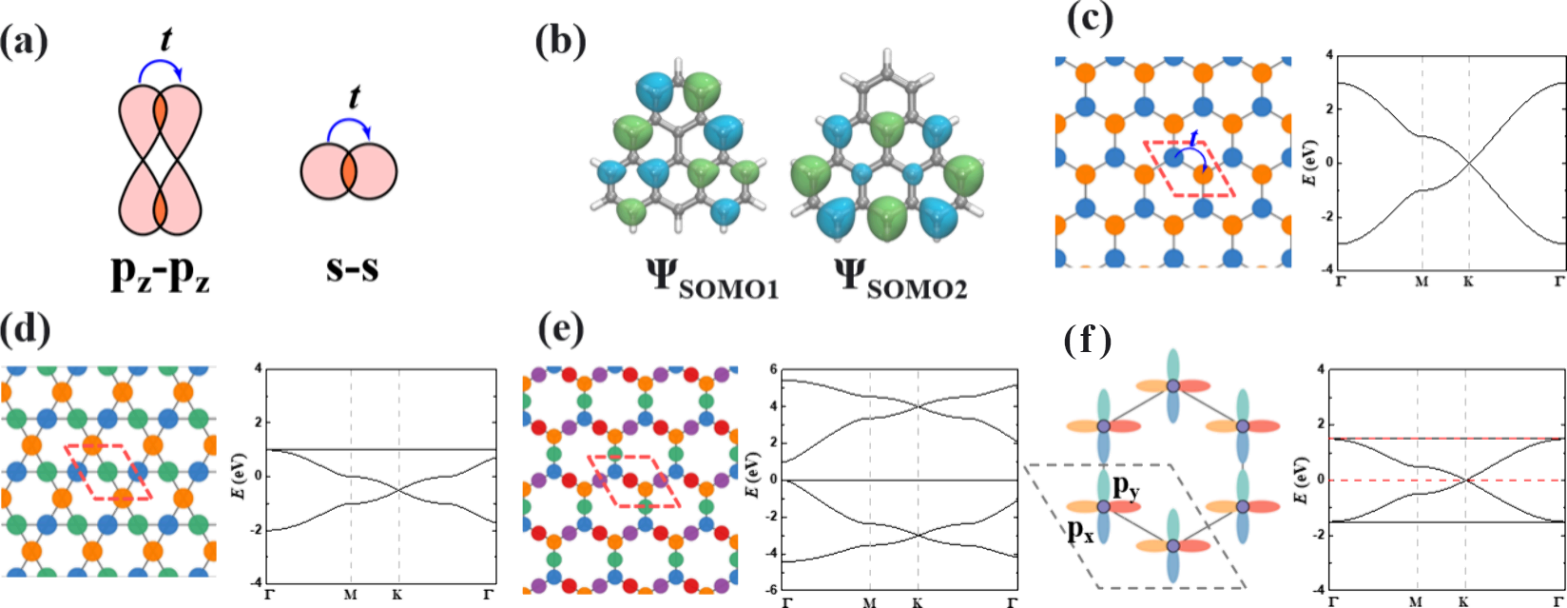
(a) Schematics of p_*z*_–p_*z*_ and s–s orbital overlaps that can be described with hopping integral *t*. (b) Two SOMOs of TRI monomer. Illustration of lattice and band structure from TB models of (c) honeycomb lattice, (d) kagome lattice, (e) honeycomb-kagome lattice, and (f) honeycomb lattice with p_*x*_-p_*y*_ orbital on each site. p_*x*_ and p_*y*_ represent the two orthogonal π orbitals (SOMOs) of the triangulene instead of individual atomic orbitals. The hopping integral *t* is shown in (c).

Triangulene (TRI), C_22_H_12_ (Figure 1a), is the polyaromatic hydrocarbon (PAH) that has been hypothesized by Erich Clar in 1953.^5^ Being a triplet diradical, its synthesis has been a challenge and was only realized after 2017 by Pavlicek et al.^6^ The TRI structure is of *D*_*3h*_ symmetry. The spin density, composed of the two single-occupied molecular orbitals (SOMOs) of the two unpaired electrons, is delocalized over the entire molecule and follows its structural symmetry. (Figures 1a and 2) Heterotriangulenes (HTs), where heteroatoms replace carbon in the center position, and/or the outer bridging carbons are substituted by functional groups (Figure 1c,d), are known since the 1970s, pioneered by Hellwinkel et al.^7−9^ Some of these systems are stable closed-shell molecules, others are monoradicals that can be stabilized as cations.

So far, extended two-dimensional (2D) structures made of covalently bound TRI or HTs have been obtained solely by on-surface synthesis employing Ullmann coupling,^10−12^ which is prone to yield small flakes and a large defect density.^13^ Recently, Contini et al. proposed a technique where hot molecules couple on top of a prestretched commensurate template, thus forming large oriented flakes, allowing for measuring their band structure features (Dirac cones and flat bands) using angle-resolved X-ray spectroscopy (ARPES).^14^ These diamagnetic 2D polymers can host intriguing electronic features such as Dirac cones.^14^ Systems with radical centers have successfully been dimerized and oligomerized, without affecting their SOMOs.^15−17^ Alternative synthesis approaches employing various coupling reactions in different environments than metal surfaces, for example at liquid/liquid or liquid/air interfaces, are explored by various groups to date.^18−21^ Many exotic properties has been predicted in these 2D systems, such as (high-order) topological insulators,^1,22^ spin frustration,^23^ Mott insulators^24^ and Stoner magnetism.^4^

In this Account, we focus on organic 2D crystals (O2DCs)^25^ made of TRI and related HTs (Figure 1). These O2DCs include the characteristic bands of the honeycomb-kagome lattice, they have the excellent optical properties of HTs, and at the same time offer a wide range of band structure engineering opportunities by incorporating heteroatoms and functional bridge groups. We will target two applications: photocatalysis and magnetism. We will further discuss the approaches that are available to theory to assess the properties of these materials, and comment on experimental challenges for synthesis and characterization.

## 2 Triangulene and Its Derivatives

TRI is the smallest nanographene and has a triplet ground state. It hosts 22 π-electrons and thus is predicted to be aromatic according to the Hückel rule. However, it is impossible to concomitantly pair all of its π-electrons into a closed-shell structure, and it therefore gives a non-Kekulé structure with two unpaired spins (Figure 1a). TRI was first synthesized on NaCl(100), Xe(111), and Cu(111) surfaces by Pavliček et al. in 2017,^6^ and in solution by Arikawa et al. in 2021.^26^ Although a crystalline structure has been obtained, three mesityl groups had to be introduced onto the γ-carbons to stabilize their radical form. Other forms of triplet TRI derivatives have been synthesized in solution.^27−29^ From its resonance configurations, TRI possesses *D*_3h_ symmetry, in accordance with the on-surface characterization using scanning tunneling and atomic force microscopy (STM, AFM).^6^ From Hückel orbital theory, TRI has two degenerate π-orbitals to host the unpaired electrons (Figure 1a). This leads to a high-spin configuration according to the Hund’s rule. The high-spin ground state is also in accord with the Ovchinnikov rule^30^ (i.e., Lieb’s theorem^31^), where, for half-filled bipartite systems, such as PAHs, the multiplicity is given by *S* = (*N*_A_ – *N*_B_)/2, where *N*_A_ and *N*_B_ are the numbers of atoms belonging to the two honeycomb sublattices A and B (Figure 1b). This relationship between the unpaired spins and sublattice imbalance is a key conclusion from the Hubbard model under half-filling conditions.^31,32^ It should be pointed out that TRI can also be referred to as [3]TRI, as other triangle-shaped nanographenes exist. The smallest one, [2]TRI, is also known as phenalenyl (PLY) and hosts one spin.^33,34^ Larger triangle-shaped nanoflakes host more unpaired electrons, whose number increases linearly with their size.^35−37^ For example, [4]TRI hosts three spins,^38^ and [5]TRI four spins, leading to *S* = 3/2 and 2 ground states, respectively^39^ (Figure 4f).

Besides triangle-shaped PAHs of various sizes, many HTs, involving B, N, O and other heteroatoms, have been predicted and synthesized.^40−42^ Most HT monomers are monoradicals or closed-shell molecules. For example, azatriangulene (TRI(N)), boratriangulene (TRI(B)), trioxatriangulene (TAM), trioxotriangulene (TOT) and trihydrotriangulene (TRIH) are monoradicals, while TAM(N), TAM(B), TOT(N), TOR(B), TRIH(B), TRIH(B) are closed-shell molecules (Figure 1). Although all before-mentioned HT radicals have delocalized spin density, TAM, TOT, and TRIH are carbon-centered radicals which, from the classic organic chemistry picture, obey the half-filling precondition of the Ovchinnikov rule. Similarly, nonplanar ring-opening derivatives, such as triphenylmethyl (TPM, or triarylmethyl) and perchlorotriphenylmethyl (PTM) radicals, are also carbon-centered radicals with delocalized spin density^43^ (Figure 1e). Introducing nitrogen or boron centers will increase or reduce the number of π-electrons, leading to fewer unpaired spins.^40,41,44−46^ Such HTs can be used as superatoms to build supramolecular architectures, such as dimers,^16,17^ nanostars,^47^ quantum rings,^48^ as well as extended structures including 1D^15^ and 2D polymers.

## 3 Tight-Binding Model of Heterotriangulene Networks

The stability and properties of triangulene and its HT derivatives are governed by their electronic π-system. As in other PAHs, it stabilizes their planar structure, dominate the structure of the frontier orbitals, and thereby their spin configuration. For symmetry reasons, the electronic π-system is decoupled from the σ-framework, which provides the bonding topology of the molecules. Numerous works have shown that Hückel theory, or its extended variant (EHT) in the case of heteroatoms, and the directly related tight-binding (TB) method, can grasp the essence of the physical and chemical interactions of such and related materials with extended π-systems, including fullerenes, nanotubes, nanoribbons, graphene, and many others. The simplicity of the method, given by its direct link between the electronic structure and the structural topology (for all-carbon π-systems, orbitals and their energies are obtained in good approximation by diagonalization of the connectivity matrix) made the method popular.

For molecular framework materials, it is convenient to coarse-grain the TB method, taking into account only a few orbitals per building block. If orbitals and the hopping parameters between the building blocks are chosen appropriately, the method offers a first, nearly quantitative, access to the band structure near the Fermi level. In the case of a vertex-transitive lattice, one orbital per lattice site is sufficient.^1^ These are formally 2p_*z*_ orbitals. As the symmetry of the system reduces the interaction to a single hopping term, which, efficiently, could also stem from the overlap between two 1s orbitals (Figure 2a). The resulting band structures for honeycomb and kagome lattices are shown in Figure 2c,d. Similarly, if two unpaired electrons are expected in the building block, (Figure 2b) it will require two TB orbitals, which can be expressed as p_*x*_ and p_*y*_ (Figure 2e). This yields the well-known band structure of the extended triangulene lattice (without consideration of spin polarization, see below) (Figure 2f).^49^ They are characterized by a Dirac point sandwiched between two flat bands.^50−53^

## 4 Electronic and Optoelectronic Properties of Diamagnetic Heterotriangulene Lattices

**Figure 3:**
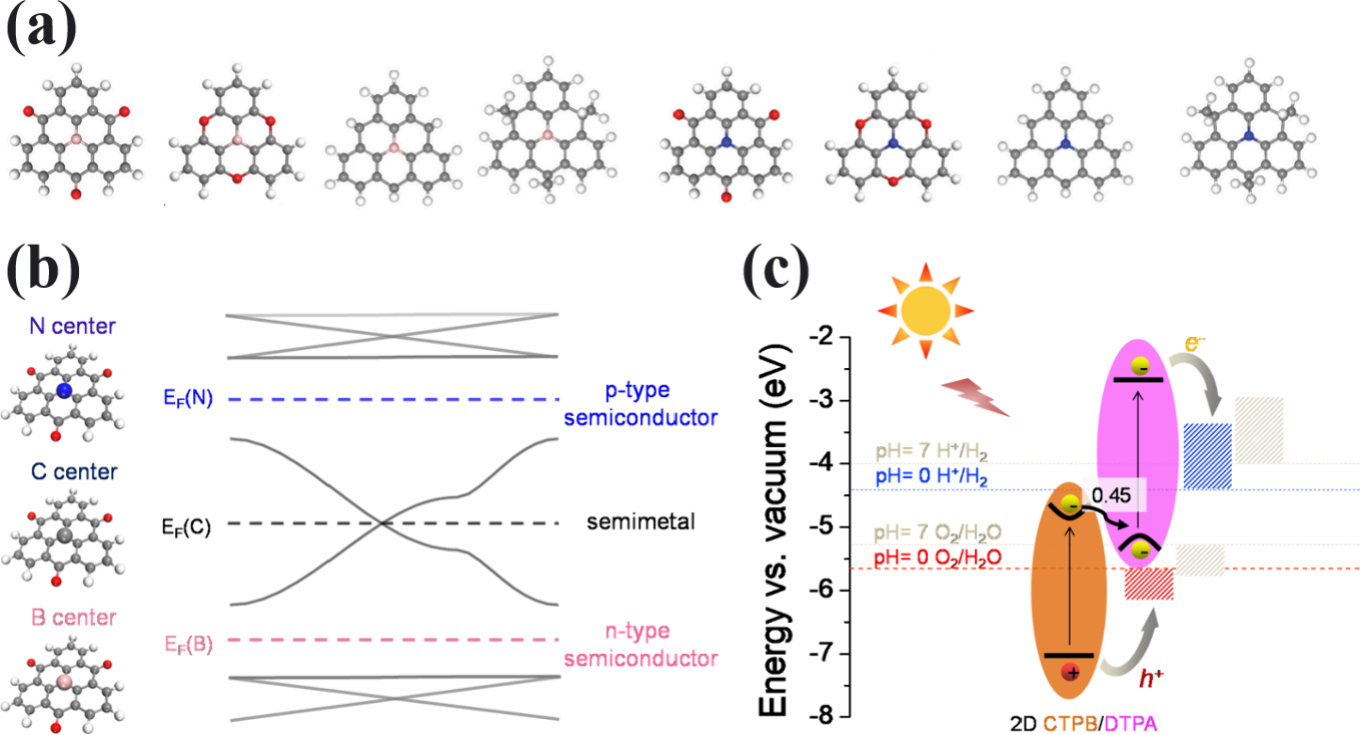
(a) Chemical structures of HT monomers for photoelectrochemical applications. Schematics for (b) the center-atom-dependent band structure of HT lattices and (c) tandem B (CTPB) and N-centered (DTPA) HT polymers for overall water splitting. When the center atom is carbon, corresponding to a half-filled π orbital of the honeycomb lattice, the Dirac point is located at the Fermi level (*E*_F_(C)). When the center atom is N or B, the Fermi level shifts upward or downward, contributing to a p- or n-type semiconductor, respectively.

The electronic structure of O2DCs, including those built of HTs (2D HT polymers), is strongly impacted by their lattice topology, which can be efficiently accessed at first sight by the TB method.^1^ The honeycomb and kagome sublattices of 2D HT polymers are the reason for the formation of Dirac cones and flat bands.^54^ The position of their Fermi level is determined by the electron configuration of the p_*z*_ orbitals of the center atoms, which are arranged in the honeycomb pattern. In a restricted calculation, a half-filled p_*z*_ orbital (carbon) contributes to the formation of a Dirac semimetal (Figure 3), whereas spin polarization opens a band gap.^24^ By contrast, an empty (boron) or a fully filled (nitrogen) p_*z*_ orbital at the HT centers leads to the formation of a n-type or p-type single-band semiconductor because of the down- or upshift of Fermi level, respectively.^55^ Analogues of hexagonal boron nitride are possible if the HT center atoms are alternating boron and nitrogen. In such systems, a band gap opens and the flat bands become dispersed, forming a direct band gap semiconductor.^56^ Although GGA-PBE provides a reliable prediction of the band dispersion, it typically underestimates the band gap. An accurate estimation of the electronic and optoelectronic properties of O2DCs requires a higher level of theory, for example, the hybrid HSE06 functional, or quasi-particle theory such as the *GW* approximation. With the potential utilization of 2D HT polymers in electronics and photocatalytic water splitting in mind, the center atoms and the bridge functional groups have been modified to tune the electronic band structure. By taking advantage of the single-band characteristics of nitrogen and boron-centered 2D HT polymers, tandem devices made of N-/B-centered HT polymers can be designed as effective photocatalysts for overall water splitting.^2^ Notably, the flat bands emerging from the kagome lattice of [4]TRI 2D polymer enable an excitonic insulator state.^57,58^

## 5 Magnetic Properties of TRI- and HT-Based Organic 2D Crystals

**Figure 4:**
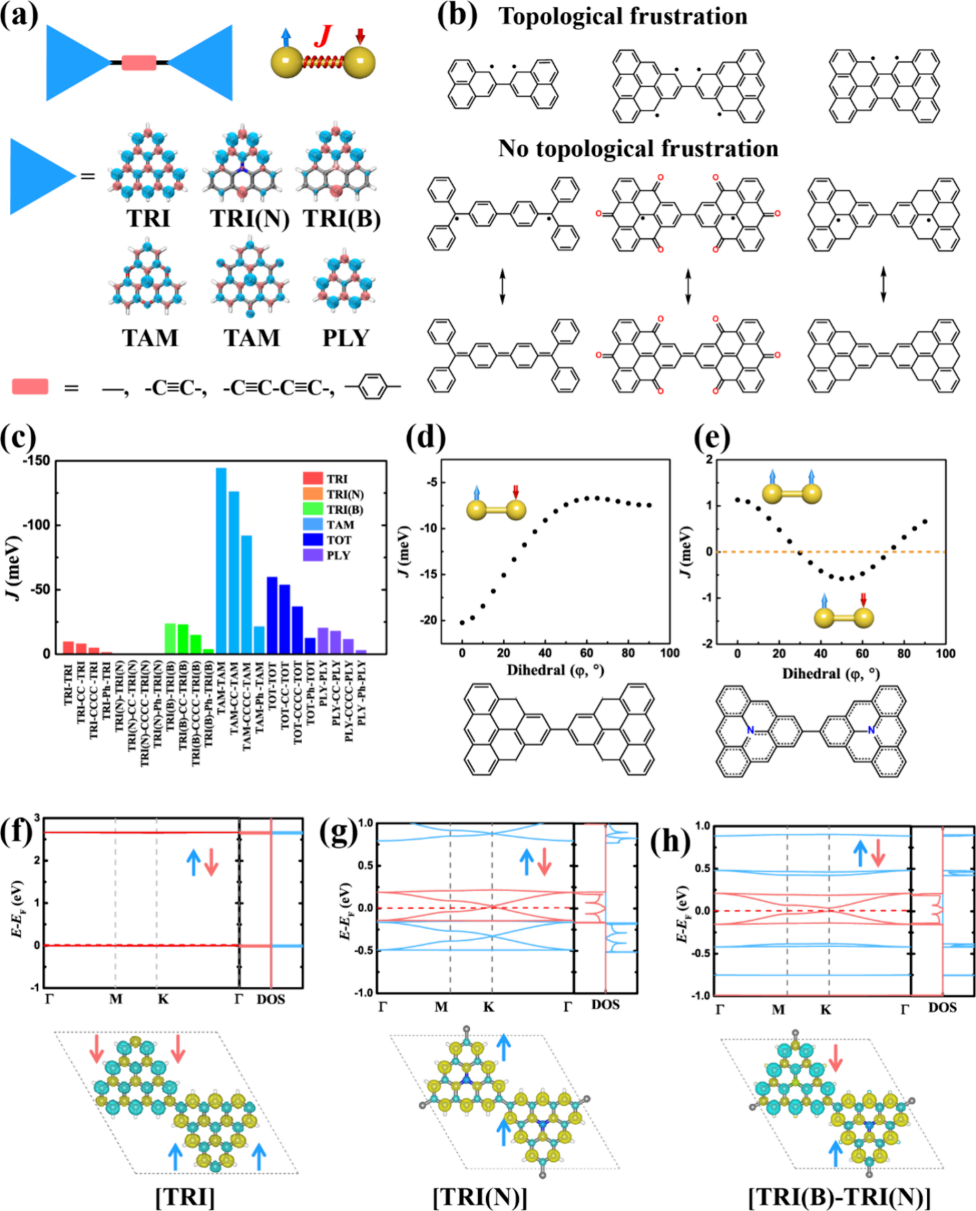
(a) Illustration of TRI-based dimers and chemical structures of the monomers as well their spin densities and linkages. (b) Topological frustration in Clar’s goblet, TRI–TRI, and PLY–PLY, compared to TPM–TPM, TAM–TAM, and TRIH–TRIH. (c) Magnetic coupling (*J*) distribution of TRI-based dimers. *J* value dependence of dihedral in the dimer for (d) TRI–TRI and (e) TRI(N)–TRI(N). Band structure and spin density of 2D polymers including (f) [TRI], (g) [TRI(N)], and (h) [TRI(B)–TRI(N)].

Integrating TRI and HT radical monomers with unpaired spins into 1D chains and 2D lattices potentially maintains spin-polarization and leads to AFM or FM magnetic order.^14,59^ In addition to the AFM spin-1 chain in TRI 1D polymers with gapped spin excitations and fractional edge states,^15^ various 1D spin-1/2 chains composed of monoradicals, such as PLY, olympicene, and diaza-hexa-peri-hexabenzocoronene, have been recently synthesized (Figure 1g). The magnetic coupling in these systems reaches values of −45 meV and demonstrate gapless excitation.^60−65^ The study of corresponding dimers offers a fascinating glimpse into the local magnetic interactions at a molecular level.^14,17,55^ These dimers, formed by linking two TRI or HT radicals through direct bonds or bridging groups such as acetylene (-C≡C-, CC), diacetylene (-C≡C–C≡C-, CCCC), or phenyl (Ph), serve as essential constructs to understand and manipulate magnetic properties for extended systems (Figure 4a). From a fundamental molecular orbital perspective, the magnetic coupling between the building units in the dimers is essentially defined by the interaction of spin-polarized orbitals. According to the Goodenough–Kanamori rule,^66,67^ the overall magnetic coupling between organic radicals is defined by two competing effects: the ferromagnetic (FM) direct exchange *K* and AFM kinetic exchange *J*_KE_ (i.e., kinetic superexchange).^68^*K* favors parallel spin alignment and high-spin configuration, arising from the direct exchange of the parallel spins in adjacent orbitals, as denied by . On the other hand, *J*_KE_ typically promotes antiparallel spin alignment in nanographene, resulting in AFM coupling and spin-polarized low-spin configuration. It originates from the virtual hopping of unpaired electrons between neighboring π-orbitals, as defined by *J*_*KE*_ = −4*t*^2^/*U*, where *t* and *U* represent the hopping integral (i.e., electronic coupling) and on-site Coulomb repulsion term in the Hubbard model.^68^ From the simplest binary picture, the delicate balance between the *K* and *J*_KE_ determines the overall magnetic coupling *J*, although other magnetic exchange mechanisms exist, such as Coulomb-driven superexchange, which can be either AFM or FM.^32,69−71^ Recently, the AFM coupling between the monomers, dominated by kinetic exchange, has been predicted in both finite and extended systems.^3,4,24,43,72−75^ 0D (dimer), 1D (spin chain) and 2D (polymer) systems with AFM coupling have been synthesized.^15−17,76,77^ Notably, AFM triangulene dimers, such as PLY–PLY and TRI–TRI, exhibit topological frustration similar to that in Clar’s goblet,^37,78,79^ whereas HT dimers do not (Figure 4b). Relevant for computer technology is the Landauer limit, i.e., the threshold for minimum energy dissipation in a state change at room temperature, which is about 18 meV.^80,81^ Enhancing AFM coupling above this limit is the condition for application in computation and data storage. One straightforward approach is to increase the electronic coupling *t* between SOMOs of monomers. However, according to the Stoner criterion, a very large *t* weakens the integrity of the spin polarization, eventually leading to a diamagnetic state when diminishing the *U*/*t* ratio.

Metal-free FM systems with high-spin configurations are unicorns in materials chemistry. From the definition of kinetic exchange, FM interaction seems to be easily achieved by reducing the orbital overlap and thus smaller *t*. However, directly reducing the overlap between the SOMOs by using nonconjugated linkages or increasing the distance between them will decrease both the direct and kinetic exchange. Alternative approaches, such as wave function phase manipulation of the spin–orbital or using the quantum interfacing effect of flat bands in 2D topology (for example in kagome and Lieb lattices), potentially produce strong FM coupling.

We will first illustrate the opportunities for rationally designing TRI and HT-based extended structures with dimers. The magnetic coupling between the SOMOs can be effectively tuned by the intrinsic properties of the radical monomer, the linkage, and the twisting angle. For half-filled systems built by hydrocarbon or carbon-centered HT radicals, open-shell singlet (OSS) ground state and AFM coupling are expected according to the Ovchinnikov rule. It is noted that SOMOs with higher symmetry generally produce stronger *J*. For example, TAM, TOT, and PLY have SOMOs of *C*_3v_ symmetry and their corresponding dimers have *J* values of −144, −60, and −20 meV, compared to TRI with SOMOs of *C*_2v_ symmetry and TRI-TRI with *J* value of −10 meV. More extended SOMO distribution will enhance the orbital overlap and therefore produce stronger *t*, resulting in stronger AFM coupling *J*, in alignment with the Goodenough–Kanamori rule (Figure 4a,c). Chemical linkages between the spin centers affect the magnetic coupling. Conjugated linkages, such as acetylene, diacetylene, vinylene, and imine, effectively mediate the electron delocalization and spin–spin interaction, compared to nonconjugated ones. However, magnetic interaction decreases rapidly with the elongation of the linkage (Figure 4c).

The twisting angle between the monomers affects conjugation and thus influences the magnetic interaction. It is noted that gas-phase dimers typically show a twisted configuration with a dihedral angle of 20–40 degrees due to steric hindrance.^3^ However, on a surface, they will adopt a planar geometry due to the strong adsorbate–substrate interaction, which is commonly seen in the experiment.^16^ Compared to the gas-phase configuration, the on-surface structures have stronger AFM coupling for carbon-centered HT dimers (Figure 4d). Among them, TAM–TAM shows the largest *J* value of about −200 meV.^3^ In contrast, the aza-triangulene dimer, TRI(N)–TRI(N) shows FM coupling at both coplanar and a largely twisted configuration, arising from the delicate balance between potential and kinetic exchange^3^ (Figure 4e). This dihedral-dependent magnetic coupling provides the foundation for exploring the origin of FM beyond the Ovchinnikov rule and the manipulation of magnetic properties via external conditions.^3,82−84^

If TRI and HT monomers are extended into periodic structures, they can form O2DCs with long-range magnetic ordering. It is noted that TRI-based O2DCs are different from graphene nanoflakes. Graphene nanoflakes can possess many degenerate π-orbitals and parallel spins within their finite structures.^37,47^ To form long-range magnetic order, they need to be used as building blocks for regular extended structures, which is similar to the relationship between transition metal atoms with partially filled d-/f-orbitals and inorganic magnets.^75^ Moreover, the bottom-up synthesis of graphene nanoflakes and magnetic 2D polymers involves different chemical reactions, where the former is the Scholl reaction and the Ullmann coupling reaction the latter. As shown in Figure 2f, TRI-based O2DCs show an exotic four-band electronic structure in the TB model, where two Dirac bands are sandwiched by two flat bands. Although the TB model without spin polarization predicts half-filled pristine TRI 2D polymer ([TRI]) to be diamagnetic with the Fermi level crossing the Dirac point, the electron correlation, specifically on-site Coulomb repulsion, opens up a large band gap of over 2.0 eV (Figure 4f), due to the large *U*/*t* radio of 17, leading to an AFM ground state with magnetic coupling of −32 meV.^4^ Similarly, other carbon-centered TRI and HT-based O2DCs also emerge as AFM Mott-Hubbard insulators.^24^

On the contrary, substituting the center atoms with boron or nitrogen as in bora- or azatriangulene shifts the Fermi level toward one of the topologically flat bands. As they have limited band dispersion and electronic coupling, the AFM kinetic exchange is suppressed, and FM potential exchange is fostered. This results in the emergence of Stoner ferromagnetism with *J* = 59 (56) meV for [TRI(N)] ([TRI(B)]). These strong FM couplings lead to Curie temperatures of 260 (250) K as demonstrated by Monte Carlo (MC) simulations. Both [TRI(N)] and [TRI(B)] show a half-metallic character, where one spin channel is semimetallic owing to the linear band crossing at the Fermi level, while the other spin channel is a single-band semiconductor (Figure 4g). This indicates their great potential to serve as spin filters and host massless spin transport. Using both aza- and boratriangulenes, one can construct a binary 2D polymer [TRI(B)-TRI(N)]. Being isoelectronic to [TRI], this system shows an *S* = 0 ground state. However, different from [TRI] with its energetically degenerate spin channel, [TRI(B)-TRI(N)] is half-semiconductor due to the different chemical potentials of different building blocks. As shown in Figure 4h, one spin channel shows a strongly opened band gap of 0.81 eV, while the other channel only has a small band gap of 37 meV. This spin-splitting behavior offers a vast opportunity to control the spin conductance in [TRI(B)-TRI(N)] via an external field. For example, using laser light with a wavelength of ∼1500 nm can excite the spin-up channel, while the other spin can be activated with increasing temperature due to the very small bandgap.^4^

For 3D magnetic systems, including stacked 1D and 2D polymers, short-range exchange between nearest neighbors can lead to infinitely long-range magnetic order according to the Mermin-Wagner theorem.^85^ However, the Mermin-Wagner theorem forbids the spontaneous breaking of continuous symmetry at finite temperature in low-dimensional systems (1D or 2D) with only short-range interactions. Similarly, due to thermal fluctuations, the Ising model rules out the existence of stable magnetic ordering for 1D systems with only short-range interactions at any finite temperature. However, long-range magnetic order has been observed in 1D systems, such as monatomic cobalt chains and the zigzag edges of narrow graphene nanoribbons.^86,87^ This discrepancy has led to a long-standing debate regarding the existence of spontaneous magnetization in low-dimensional systems, especially in metal-free systems with small spin–orbit coupling and low magnetic anisotropy. Recent MC simulations demonstrate that for finite systems stable magnetic ordering based on short-range interactions are possible,^88^ with a maximum system size corresponding to the spin–spin correlation length which extends over several millimeters. Another argument suggests that long-range magnetic interactions, such as dipole–dipole interactions, Coulomb interactions, or Ruderman–Kittel–Kasuya–Yosida (RKKY) interactions, could stabilize long-range magnetic order, while still complying with the conditions of the Mermin–Wagner theorem.^89^

## 6 Challenges for Theory

**Figure 5:**
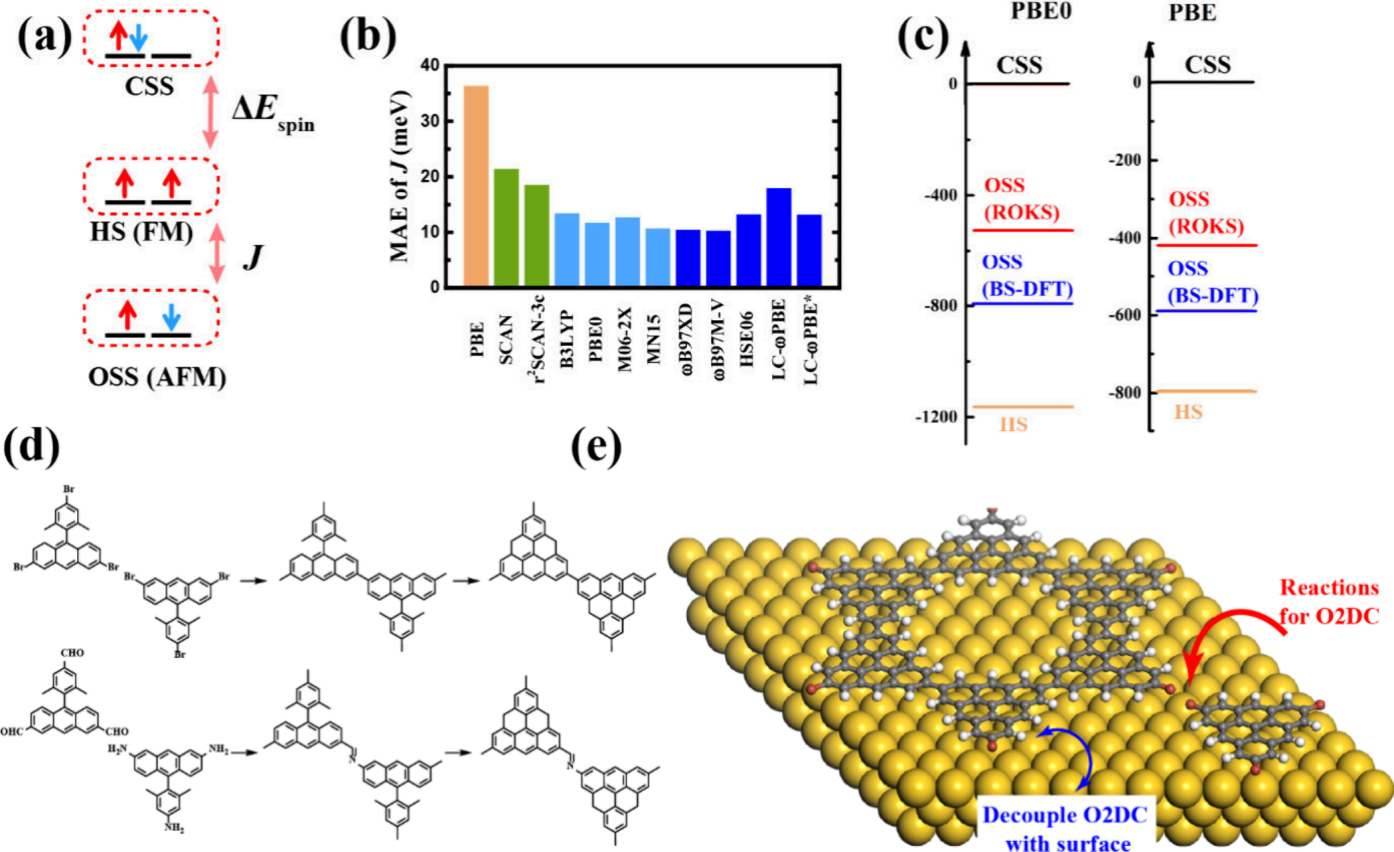
(a) Illustration of the multireference problem of metal-free magnetism. The closed-shell singlet (CSS), high-spin (HS), and open-shell singlet states (OSS) are shown. (b) Mean average error (MAE) of BS-DFT for magnetic coupling *J* with various functionals in a TRI/HT-based molecular data set. (c) Relative energies (*E* (meV)) of the CSS, HS, and OSS states (calculated by the ROKS and BS-DFT approaches) for triangulene monomer (TRI) with triplet ground state as implemented in Q-Chem 5.3.^100^ The basis set is def2-TZVP. (d) Schematic diagram of Ullmann coupling and Schiff base polycondensation for TRI-based O2DC. (e) Illustration of on-surface synthesis and coupling between the O2DC and Au(111) surface.

Predicting the magnetic properties of open-shell TRI-based systems is essentially a multireference (MR) problem, due to many nearly degenerate states including FM, AFM and diamagnetic states (Figure 5a). Multiconfigurational approaches in principle are required to include both the static and dynamic correlations. Recently, the complete active space self-consistent field/n-electron valence second-order perturbation theory (CASSCF/NEVPT2) method has been used to investigate the magnetic coupling of TRI-based molecular systems and reaches good agreement with experiment.^90^ One potential shortcoming of CASSCF is that the active space with today’s computational resources is limited to 16 orbitals due to the large computational demand of full configuration interaction (FCI) within the active space.^91^ Therefore, these approaches are not suitable for systems with more unpaired electrons or degenerate π-orbitals. The density matrix renormalization group (DMRG) method could be a more potential solution to include more orbitals into the active space,^92−94^ however, reducing the computational demand and including dynamic correlation is still challenging. Another issue is that most multireference methods can only be applied to finite systems, and have not been implemented to solve magnetic systems with extended structures. This poses a severe challenge in studying the magnetic properties of periodic systems. DFT provides an efficient approach to study the magnetic behavior of large and extended systems.^95,96^ Kohn–Sham (KS)-DFT is essentially a single-reference method, and, for an electronic state that is dominated by a single determinant, such as the high-spin (HS) state, unrestricted (U) DFT works well for describing the electronic structure and magnetic properties. However, in order to calculate magnetic couplings and energy diagrams, the AFM open-shell singlet state is always necessary, but cannot be well described by a single-reference method in principle. How to properly include a multireference feature into the DFT framework is still an open question. For strongly correlated systems with transition metal atoms where the correlation is dominated by degenerate d-orbitals, the DFT+U approach, with the empirical Hubbard U parameter, is a popular solution to describe magnetism. However, for metal-free systems, it is impossible to attribute the static electron correlation to a well-defined subset of orbitals and thus the DFT+U method is inappropriate. The broken-symmetry (BS) approach provides an alternative to describe the OSS state. It can be used to predict the ground state and the magnetic coupling with qualitative accuracy, although the accuracy largely depends on the density functional and mapping approaches. From a systematic benchmark study, PBE0, M06-2X, and MN15 are verified for TRI-based molecules to predict the *J* value with a small mean absolute error of 12, 13, and 11 meV, respectively^90^ (Figure 5b). One point constantly argued for BS-DFT is the severe spin contamination, especially for describing the OSS state, where the solution is a singlet–triplet mixture. Many efforts have been devoted to remove the spin contamination of the OSS state, although the physical interpretation for the expectation value of the many-electron spin operator, < *S*^2^> in KS-DFT is unclear.^95^ One possible approach is to employ restricted open-shell KS DFT (ROKS), as it integrates some multireference character into the calculation.^97^ It simultaneously optimizes the excited singlet (i.e., mixed state) and triplet state and produces the total energy without spin contamination by *E* (OSS state) = 2*E*(mixed state) – *E*(triplet state).^97^ This method has been highly successful in predicting excitation energies for charge-transfer states and core excitations.^98,99^ However, it may overestimate the *J* values, as observed, for example, in TRI monomer (Figure 5c), due to the absence of double excitation, which is physically relevant for assessing the superexchange interaction. Although the ROKS approach is very useful for describing the OSS excited state where the triplet state has lowest energy, it does not apply to the OSS ground state or multiradicals, such as [5] TRI, as only one electron pair is allowed to be broken. Therefore, a unified approach to predict and describe systems with both a high-spin and a low-spin ground state is necessary. Further theory development is required to allow for the correct description of metal-free magnetism in extended systems.

## 7 Challenges for Experiment

Synthetic strategies for TRI-based O2DCs with long-range ordering are another challenge, especially for those with radicaloid building units. Solvent-free on-surface reactions, such as Ullmann and Yamaguchi couplings, offer an attractive approach for synthesizing O2DCs with atomic precision, where the surfaces serve as the template, interface, and catalyst.^13^ However, due to the lack of reversibility of these reactions and the limited mobility of absorbed monomers on the surface, realizing large-scale, defect-free, and high-quality 2D polymers is very challenging.^13^ Although rapid progress has been achieved in the synthesis of magnetic 1D polymers and nanoribbons as well as diamagnetic 2D polymers,^14,15,60−65,101−103^ only small magnetic 2D structures of [4]TRI have been synthesized to date.^104^ It will be also promising to explore the reticular chemistry reaction commonly used to form covalent organic frameworks (COFs), such as Knoevenagel polycondensation, Wittig reaction, and Schiff-base polycondensation, where chemical equilibrium at the liquid–liquid and liquid–solid and interface are manipulated (Figure 5d). These in-solution polymerization reactions potentially provide better processability and crystallinity of the sample. While diamagnetic TRI-based 2D polymers have been realized,^14,59^ magnetic 2D polymers are still very rare,^12^ probably due to the active reactivity of radical units and twisted geometry of the precursor impeding the assembling and growing of well-ordered 2D lattice. Although many exotic physical phenomena have been predicted in magnetic TRI-based O2DCs, making the 2D lattice with atomically precise structures and directly characterizing the magnetic properties requires further effort. Specifically, the strong interaction between the magnetic O2DCs and metal surfaces, such as Ag(111) and Au(111), severely influences the direct measurement of intrinsic properties (Figure 5e). For example, if there is a Fermi level mismatch between the metal surface and 2D polymer, the charge transfer could happen and may quench or enhance the magnetic moments.^40^ Many approaches have been proposed to decouple the O2DCs from the metal surface, including the use of a semiconducting substrate, such as TiO_2_(011) and Al_2_O_3_, or an intercalating layer.^105,106^ Moreover, the interaction between magnetic O2DCs and a metal surface can induce the Kondo effect, where the itinerant electrons of the metal screen the localized spins of the 2D polymers, thereby complicating the direct observation of these magnetic properties.

## 8 Conclusions and Perspectives

Over the past few years, significant progress in both experiment and theory of triangulene-based molecules and organic 2D crystals has been achieved. The diversity of building blocks provides a vast chemical space to explore the great tunability in optoelectronic and magnetic properties. These atomically precise 2D lattices provide a promising platform to explore novel electronic structures and exotic physical phenomena, such as Dirac cones, flat bands, Stoner and Mott-Hubbard magnetism, spin liquids, topological insulators, and so forth. The research of TRI-based O2DCs is still at its early stage and this fundamental research provides a solid foundation for potential applications in organic optoelectronic and spintronics.

Apart from the remarkable advances, several aspects remain to be explored:

(1) General strategies to fabricate large-scale and high-quality (hetero)triangulene-based O2DCs at various interfaces require to be explored. This is of paramount importance for directly characterizing their novel chemical and physical properties. The ability to consistently produce these materials at scale will open up new avenues for both fundamental research and technological innovation.
(2) The impact of stacking of individual 2D polymer layers should be investigated. Particularly, the stacking mode of magnetic 2D polymers could significantly affect their out-of-plane coupling.^107^ Understanding these interactions could lead to the development of materials with tunable magnetic properties, potentially revolutionizing fields such as data storage and quantum computing.
(3) Beyond individual 2D polymers and homogeneously stacked structures, it would be of great interest to explore heterostructures formed by inorganic substrates, especially with magnetic 2D materials, such as CrI_3_, NiPS_3_, and Cr_2_Ge_2_Te_6_. These hybrid structures could exhibit novel phenomena arising from the interplay between organic and inorganic components, potentially leading to unprecedented magnetic, electronic, or optical properties.
(4) Exploring methods to dynamically control the properties of these materials through external stimuli such as light, electric fields, or mechanical strain could open up exciting possibilities for responsive and adaptive devices.

## References

[ref1] Springer M. A.; Liu T.-J.; Kuc A.; Heine T. Topological two-dimensional polymers. Chem. Soc. Rev. 2020, 49 (7), 2007–2019. 10.1039/C9CS00893D. 32206766

[ref2] Jing Y.; Zhou Z.; Geng W.; Zhu X.; Heine T. 2D Honeycomb-Kagome Polymer Tandem as Effective Metal-Free Photocatalysts for Water Splitting. Adv. Mater. 2021, 33 (21), e2008645 10.1002/adma.202008645. 33942398 PMC11468641

[ref3] Yu H.; Heine T. Magnetic Coupling Control in Triangulene Dimers. J. Am. Chem. Soc. 2023, 145 (35), 19303–19311. 10.1021/jacs.3c05178. 37610306 PMC10485925

[ref4] Yu H.; Heine T. Prediction of metal-free Stoner and Mott-Hubbard magnetism in triangulene-based two-dimensional polymers. Sci. Adv. 2024, 10 (40), eadq7954 10.1126/sciadv.adq7954. 39356753 PMC12697527

[ref5] Clar E.; Stewart D. G. Aromatic hydrocarbons. LXV. Triangulene derivatives1. J. Am. Chem. Soc. 1953, 75 (11), 2667–2672. 10.1021/ja01107a035.

[ref6] Pavliček N.; Mistry A.; Majzik Z.; Moll N.; Meyer G.; Fox D. J.; Gross L. Synthesis and characterization of triangulene. Nature Nanotechnol. 2017, 12 (4), 308–311. 10.1038/nnano.2016.305. 28192389

[ref7] Hellwinkel D.; Melan M. Heteropolycyclen vom Triangulen-Typ, I. 8.12-Dihydro-4H-benzo[1.9]chinolizino[3.4.5.6.7- defg ]acridin-trion-(4.8.12) und 5.9-Dihydro-chino[3.2.1- de ]acridin-dion-(5.9). Chem. Ber. 1971, 104 (4), 1001–1016. 10.1002/cber.19711040406.

[ref8] Hellwinkel D.; Melan M. Heteropolycyclen vom Triangulen-Typ, II. Zur Stereochemie verbrückter Triarylamine. Chem. Ber. 1974, 107 (2), 616–626. 10.1002/cber.19741070231.

[ref9] Hellwinkel D.; Schmidt W. Modifizierte Tetrahelicen-Systeme, III. Zweifach ortho -verbrückte Triphenylamin-Derivate. Chem. Ber. 1980, 113 (1), 358–384. 10.1002/cber.19801130136.

[ref10] Bieri M.; Blankenburg S.; Kivala M.; Pignedoli C. A.; Ruffieux P.; Müllen K.; Fasel R. Surface-supported 2D heterotriangulene polymers. Chemical communications (Cambridge, England) 2011, 47 (37), 10239–10241. 10.1039/c1cc12490k. 21850288

[ref11] Schlütter F.; Rossel F.; Kivala M.; Enkelmann V.; Gisselbrecht J.-P.; Ruffieux P.; Fasel R.; Müllen K. π-Conjugated heterotriangulene macrocycles by solution and surface-supported synthesis toward honeycomb networks. J. Am. Chem. Soc. 2013, 135 (11), 4550–4557. 10.1021/ja400857g. 23437809

[ref12] Steiner C.; Gebhardt J.; Ammon M.; Yang Z.; Heidenreich A.; Hammer N.; Görling A.; Kivala M.; Maier S. Hierarchical on-surface synthesis and electronic structure of carbonyl-functionalized one- and two-dimensional covalent nanoarchitectures. Nat. Commun. 2017, 8, 14765 10.1038/ncomms14765. 28322232 PMC5364392

[ref13] Jing Y.; Zhu X.; Maier S.; Heine T. 2D conjugated polymers: exploiting topological properties for the rational design of metal-free photocatalysts. Trends in Chemistry 2022, 4 (9), 792–806. 10.1016/j.trechm.2022.06.002.

[ref14] Galeotti G.; De Marchi F.; Hamzehpoor E.; MacLean O.; Rajeswara Rao M.; Chen Y.; Besteiro L. V.; Dettmann D.; Ferrari L.; Frezza F.; Sheverdyaeva P. M.; Liu R.; Kundu A. K.; Moras P.; Ebrahimi M.; Gallagher M. C.; Rosei F.; Perepichka D. F.; Contini G. Synthesis of mesoscale ordered two-dimensional π-conjugated polymers with semiconducting properties. Nature materials 2020, 19 (8), 874–880. 10.1038/s41563-020-0682-z. 32424372

[ref15] Mishra S.; Catarina G.; Wu F.; Ortiz R.; Jacob D.; Eimre K.; Ma J.; Pignedoli C. A.; Feng X.; Ruffieux P.; et al. Observation of fractional edge excitations in nanographene spin chains. Nature 2021, 598 (7880), 287–292. 10.1038/s41586-021-03842-3. 34645998

[ref16] Mishra S.; Beyer D.; Eimre K.; Ortiz R.; Fernández-Rossier J.; Berger R.; Gröning O.; Pignedoli C. A.; Fasel R.; Feng X.; Ruffieux P. Collective all-carbon magnetism in triangulene dimers. Angew. Chem. 2020, 132 (29), 12139–12145. 10.1002/ange.202002687. PMC7383983 32301570

[ref17] Krane N.; Turco E.; Bernhardt A.; Jacob D.; Gandus G.; Passerone D.; Luisier M.; Juríček M.; Fasel R.; Fernández-Rossier J.; Ruffieux P. Exchange interactions and intermolecular hybridization in a spin-1/2 nanographene dimer. Nano Lett. 2023, 23 (20), 9353–9359. 10.1021/acs.nanolett.3c02633. 37819646

[ref18] Li Z.; He T.; Gong Y.; Jiang D. Covalent organic frameworks: pore design and interface engineering. Acc. Chem. Res. 2020, 53 (8), 1672–1685. 10.1021/acs.accounts.0c00386. 32786335

[ref19] Lohse M. S.; Bein T. Covalent organic frameworks: structures, synthesis, and applications. Adv. Funct. Mater. 2018, 28 (33), 1705553 10.1002/adfm.201705553.

[ref20] Ni F.; Wang Z.; Feng X. On-Water Surface Synthesis of Two-Dimensional Polymer Membranes for Sustainable Energy Devices. Acc. Chem. Res. 2024, 57, 2414 10.1021/acs.accounts.4c00356. 39126386 PMC11339920

[ref21] Zhang T.; Zhang G.; Chen L. 2D conjugated covalent organic frameworks: defined synthesis and tailor-made functions. Acc. Chem. Res. 2022, 55 (6), 795–808. 10.1021/acs.accounts.1c00693. 35025209

[ref22] Ni X.; Huang H.; Brédas J.-L. Organic higher-order topological insulators: heterotriangulene-based covalent organic frameworks. J. Am. Chem. Soc. 2022, 144 (49), 22778–22786. 10.1021/jacs.2c11229. 36469524

[ref23] Alcón I.; Ribas-Ariño J.; Moreira I. d. P.; Bromley S. T. Emergent spin frustration in neutral mixed-valence 2D conjugated polymers: A potential quantum materials platform. J. Am. Chem. Soc. 2023, 145 (10), 5674–5683. 10.1021/jacs.2c11185. 36877195 PMC10021012

[ref24] Thomas S.; Li H.; Bredas J.-L. Emergence of an antiferromagnetic Mott insulating phase in hexagonal π-conjugated covalent organic frameworks. Adv. Mater. 2019, 31 (17), 1900355 10.1002/adma.201900355. 30847999

[ref25] Wang Z.; Wang M.; Heine T.; Feng X. Electronic and quantum properties of organic two-dimensional crystals. Nat. Rev. Mater. 2024, 10.1038/s41578-024-00740-8.

[ref26] Arikawa S.; Shimizu A.; Shiomi D.; Sato K.; Shintani R. Synthesis and isolation of a kinetically stabilized crystalline triangulene. J. Am. Chem. Soc. 2021, 143 (46), 19599–19605. 10.1021/jacs.1c10151. 34767718

[ref27] Inoue J.; Fukui K.; Kubo T.; Nakazawa S.; Sato K.; Shiomi D.; Morita Y.; Yamamoto K.; Takui T.; Nakasuji K. The first detection of a Clar’s hydrocarbon, 2, 6, 10-tri-tert-butyltriangulene: a ground-state triplet of non-Kekulé polynuclear benzenoid hydrocarbon. J. Am. Chem. Soc. 2001, 123 (50), 12702–12703. 10.1021/ja016751y. 11741445

[ref28] Allinson G.; Bushby R. J.; Paillaud J. L.; Oduwole D.; Sales K. ESR spectrum of a stable triplet. pi. biradical: trioxytriangulene. J. Am. Chem. Soc. 1993, 115 (5), 2062–2064. 10.1021/ja00058a076.

[ref29] Allinson G.; Bushby R. J.; Paillaud J.-L.; Thornton-Pett M. Synthesis of a derivative of triangulene; the first non-kekulé polynuclear aromatic. Journal of the Chemical Society, Perkin Transactions 1 1995, (4), 385–390. 10.1039/P19950000385.

[ref30] Ovchinnikov A. A. Multiplicity of the ground state of large alternant organic molecules with conjugated bonds: (Do Organic Ferromagnetics Exist?). Theoretica Chimica Acta 1978, 47, 297–304. 10.1007/BF00549259.

[ref31] Lieb E. H. Two theorems on the Hubbard model. Phys. Rev. Lett. 1989, 62 (10), 1201 10.1103/PhysRevLett.62.1201. 10039602

[ref32] Ortiz R.; Boto R. A.; García-Martínez N.; Sancho-Garcia J. C.; Melle-Franco M.; Fernández-Rossier J. Exchange rules for diradical π-conjugated hydrocarbons. Nano Lett. 2019, 19 (9), 5991–5997. 10.1021/acs.nanolett.9b01773. 31365266

[ref33] Turco E.; Bernhardt A.; Krane N.; Valenta L.; Fasel R.; Juríček M.; Ruffieux P. Observation of the magnetic ground state of the two smallest triangular nanographenes. JACS Au 2023, 3 (5), 1358–1364. 10.1021/jacsau.2c00666. 37234116 PMC10207087

[ref34] Goto K.; Kubo T.; Yamamoto K.; Nakasuji K.; Sato K.; Shiomi D.; Takui T.; Kubota M.; Kobayashi T.; Yakusi K.; Ouyang J. A stable neutral hydrocarbon radical: synthesis, crystal structure, and physical properties of 2, 5, 8-tri-tert-butyl-phenalenyl. J. Am. Chem. Soc. 1999, 121 (7), 1619–1620. 10.1021/ja9836242.

[ref35] Mishra S.; Xu K.; Eimre K.; Komber H.; Ma J.; Pignedoli C. A.; Fasel R.; Feng X.; Ruffieux P. Synthesis and characterization of [7] triangulene. Nanoscale 2021, 13 (3), 1624–1628. 10.1039/D0NR08181G. 33443270

[ref36] Su J.; Telychko M.; Song S.; Lu J. Triangulenes: from precursor design to on-surface synthesis and characterization. Angew. Chem., Int. Ed. 2020, 59 (20), 7658–7668. 10.1002/anie.201913783. 31872494

[ref37] Wang W. L.; Meng S.; Kaxiras E. Graphene nanoflakes with large spin. Nano Lett. 2008, 8 (1), 241–245. 10.1021/nl072548a. 18052302

[ref38] Mishra S.; Beyer D.; Eimre K.; Liu J.; Berger R.; Gröning O.; Pignedoli C. A.; Müllen K.; Fasel R.; Feng X.; Ruffieux P. Synthesis and characterization of π-extended triangulene. J. Am. Chem. Soc. 2019, 141 (27), 10621–10625. 10.1021/jacs.9b05319. 31241927

[ref39] Su J.; Telychko M.; Hu P.; Macam G.; Mutombo P.; Zhang H.; Bao Y.; Cheng F.; Huang Z.-Q.; Qiu Z.; et al. Atomically precise bottom-up synthesis of π-extended [5] triangulene. Sci. Adv. 2019, 5 (7), eaav7717 10.1126/sciadv.aav7717. 31360763 PMC6660211

[ref40] Wang T.; Berdonces-Layunta A.; Friedrich N.; Vilas-Varela M.; Calupitan J. P.; Pascual J. I.; Peña D.; Casanova D.; Corso M.; de Oteyza D. G. Aza-triangulene: On-surface synthesis and electronic and magnetic properties. J. Am. Chem. Soc. 2022, 144 (10), 4522–4529. 10.1021/jacs.1c12618. 35254059 PMC8931755

[ref41] Wei H.; Hou X.; Xu T.; Zou Y.; Li G.; Wu S.; Geng Y.; Wu J. Solution-Phase Synthesis and Isolation of An Aza-Triangulene and Its Cation in Crystalline Form. Angew. Chem. 2022, 134 (40), e202210386 10.1002/ange.202210386. 36000462

[ref42] Hirai M.; Tanaka N.; Sakai M.; Yamaguchi S. Structurally constrained boron-, nitrogen-, silicon-, and phosphorus-centered polycyclic π-conjugated systems. Chem. Rev. 2019, 119 (14), 8291–8331. 10.1021/acs.chemrev.8b00637. 30860363

[ref43] Alcón I.; Santiago R.; Ribas-Arino J.; Deumal M.; Moreira I. d. P.; Bromley S. T. Controlling pairing of π-conjugated electrons in 2D covalent organic radical frameworks via in-plane strain. Nat. Commun. 2021, 12 (1), 1705 10.1038/s41467-021-21885-y. 33731706 PMC7969611

[ref44] Calupitan J. P.; Berdonces-Layunta A.; Aguilar-Galindo F.; Vilas-Varela M.; Peña D.; Casanova D.; Corso M.; de Oteyza D. G.; Wang T. Emergence of π-Magnetism in Fused Aza-Triangulenes: Symmetry and Charge Transfer Effects. Nano Lett. 2023, 23 (21), 9832–9840. 10.1021/acs.nanolett.3c02586. 37870305 PMC10722538

[ref45] Lawrence J.; He Y.; Wei H.; Su J.; Song S.; Wania Rodrigues A.; Miravet D.; Hawrylak P.; Zhao J.; Wu J.; Lu J. Topological Design and Synthesis of High-Spin Aza-triangulenes without Jahn–Teller Distortions. ACS Nano 2023, 17 (20), 20237–20245. 10.1021/acsnano.3c05974. 37791737

[ref46] Vilas-Varela M.; Romero-Lara F.; Vegliante A.; Calupitan J. P.; Martínez A.; Meyer L.; Uriarte-Amiano U.; Friedrich N.; Wang D.; Schulz F.; et al. On-Surface Synthesis and Characterization of a High-Spin Aza-[5]-Triangulene. Angew. Chem. 2023, 135 (41), e202307884 10.1002/ange.202307884. 37604782

[ref47] Hieulle J.; Castro S.; Friedrich N.; Vegliante A.; Lara F. R.; Sanz S.; Rey D.; Corso M.; Frederiksen T.; Pascual J. I.; Peña D. On-surface synthesis and collective spin excitations of a triangulene-based nanostar. Angew. Chem., Int. Ed. 2021, 60 (48), 25224–25229. 10.1002/anie.202184861. PMC9292598 34647398

[ref48] Su J.; Fan W.; Mutombo P.; Peng X.; Song S.; Ondráček M.; Golub P.; Brabec J.; Veis L.; Telychko M.; et al. On-surface synthesis and characterization of [7] triangulene quantum ring. Nano Lett. 2021, 21 (1), 861–867. 10.1021/acs.nanolett.0c04627. 33305570

[ref49] Jiang W.; Ni X.; Liu F. Exotic topological bands and quantum states in metal–organic and covalent–organic frameworks. Acc. Chem. Res. 2021, 54 (2), 416–426. 10.1021/acs.accounts.0c00652. 33400497

[ref50] Wu C.; Bergman D.; Balents L.; Das Sarma S. Flat bands and Wigner crystallization in the honeycomb optical lattice. Phys. Rev. Lett. 2007, 99 (7), 070401 10.1103/PhysRevLett.99.070401. 17930875

[ref51] Anindya K. N.; Rochefort A. Controlling the magnetic properties of two-dimensional carbon-based Kagome polymers. Carbon Trends 2022, 7, 100170 10.1016/j.cartre.2022.100170.

[ref52] Kan E.; Hu W.; Xiao C.; Lu R.; Deng K.; Yang J.; Su H. Half-metallicity in organic single porous sheets. J. Am. Chem. Soc. 2012, 134 (13), 5718–5721. 10.1021/ja210822c. 22440006

[ref53] Spalenza P. E. P.; de Souza F. A. L.; Amorim R. G.; Scheicher R. H.; Scopel W. L. A high density nanopore 3-triangulene kagome lattice. Nanoscale 2024, 16 (20), 9911–9916. 10.1039/D4NR00910J. 38686534

[ref54] Jing Y.; Heine T. Making 2D topological polymers a reality. Nat. Mater. 2020, 19 (8), 823–824. 10.1038/s41563-020-0690-z. 32424370

[ref55] Jing Y.; Heine T. Two-Dimensional Kagome Lattices Made of Hetero Triangulenes Are Dirac Semimetals or Single-Band Semiconductors. J. Am. Chem. Soc. 2019, 141 (2), 743–747. 10.1021/jacs.8b09900. 30499300

[ref56] Zhou Z.; Springer M. A.; Geng W.; Zhu X.; Li T.; Li M.; Jing Y.; Heine T. Rational Design of Two-Dimensional Binary Polymers from Heterotriangulenes for Photocatalytic Water Splitting. J. Phys. Chem. Lett. 2021, 12 (33), 8134–8140. 10.1021/acs.jpclett.1c02109. 34410139

[ref57] Sethi G.; Zhou Y.; Zhu L.; Yang L.; Liu F. Flat-band-enabled triplet excitonic insulator in a diatomic kagome lattice. Phys. Rev. Lett. 2021, 126 (19), 196403 10.1103/PhysRevLett.126.196403. 34047585

[ref58] Sethi G.; Cuma M.; Liu F. Excitonic condensate in flat valence and conduction bands of opposite chirality. Phys. Rev. Lett. 2023, 130 (18), 186401 10.1103/PhysRevLett.130.186401. 37204894

[ref59] Dettmann D.; Sheverdyaeva P. M.; Hamzehpoor E.; Franchi S.; Galeotti G.; Moras P.; Ceccarelli C.; Perepichka D. F.; Rosei F.; Contini G. Electronic Band Engineering of Two-Dimensional Kagome Polymers. ACS Nano 2024, 18 (1), 849–857. 10.1021/acsnano.3c09476. 38147033

[ref60] Yuan Z.; Zhang X.-Y.; Jiang Y.; Qian X.; Wang Y.; Liu Y.; Liu L.; Liu X.; Guan D.; Li Y.; Zheng H.; Liu C.; Jia J.; Qin M.; Liu P.-N.; Li D.-Y.; Wang S. Atomic-Scale Imaging of Fractional Spinon Quasiparticles in Open-Shell Triangulene Spin-1/2 Chains. arXiv preprint 2024, arXiv:2408.08612 10.48550/arXiv.2408.0861. 39874540

[ref61] Fu X.; Huang L.; Liu K.; Henriques J. C. G.; Gao Y.; Han X.; Chen H.; Wang Y.; Palma C.-A.; Cheng Z.; Lin X.; Du Du S.; Ma J.; Fernández-Rossier J.; Feng X.; Gao H.-J. Building spin-1/2 antiferromagnetic Heisenberg chains with diaza-nanographenes. arXiv preprint 2024, arXiv:2407.20511 10.48550/arXiv.2407.20511.

[ref62] Zhao C.; Yang L.; Henriques J. C. G.; Ferri-Cortés M.; Catarina G.; Pignedoli C. A.; Ma J.; Feng X.; Ruffieux P.; Fernández-Rossier J.; Fasel R. Gapless spin excitations in nanographene-based antiferromagnetic spin-1/2 Heisenberg chains. arXiv preprint 2024, arXiv:2408.10045 10.48550/arXiv.2408.10045. PMC12048352 40087538

[ref63] Su X.; Ding Z.; Hong Y.; Ke N.; Yan K.; Li C.; Jiang Y.; Yu P. Fabrication of Spin-1/2 Heisenberg Antiferromagnetic Chains via Combined On-surface Synthesis and Reduction for Spinon Detection. arXiv preprint 2024, arXiv:2408.08801 10.48550/arXiv.2408.08801.

[ref64] Sun K.; Cao N.; Silveira O. J.; Fumega A. O.; Hanindita F.; Ito S.; Lado J. L.; Liljeroth P.; Foster A. S.; Kawai S. Heisenberg Spin-1/2 Antiferromagnetic Molecular Chains. arXiv preprint 2024, arXiv:2407.02142 10.48550/arXiv.2407.02142. PMC11870052 40020073

[ref65] Zhao C.; Catarina G.; Zhang J.-J.; Henriques J. C. G.; Yang L.; Ma J.; Feng X.; Gröning O.; Ruffieux P.; Fernández-Rossier J.; Fasel R. Tunable topological phases in nanographene-based spin-1/2 alternating-exchange Heisenberg chains. Nature Nanotechnol. 2024, 10.1038/s41565-024-01805-z. 39468357

[ref66] Kanamori J. Superexchange interaction and symmetry properties of electron orbitals. J. Phys. Chem. Solids 1959, 10 (2–3), 87–98. 10.1016/0022-3697(59)90061-7.

[ref67] Goodenough J. B. Theory of the role of covalence in the perovskite-type manganites [La, M (II)] MnO_3_. Phys. Rev. 1955, 100 (2), 564 10.1103/PhysRev.100.564.

[ref68] Mori T. Electronic properties of organic conductors; Springer, 2016.

[ref69] Henriques J. C. G.; Fernández-Rossier J. Anatomy of linear and nonlinear intermolecular exchange in *S* = 1 nanographene. Phys. Rev. B 2023, 108 (15), 155423 10.1103/PhysRevB.108.155423.

[ref70] Jacob D.; Fernández-Rossier J. Theory of intermolecular exchange in coupled spin-1/2 nanographenes. Phys. Rev. B 2022, 106 (20), 205405 10.1103/PhysRevB.106.205405.

[ref71] Saleem Y.; Steenbock T.; Alhadi E. R. J.; Pasek W.; Bester G.; Potasz P. Superexchange Mechanism in Coupled Triangulenes Forming Spin-1 Chains. Nano Lett. 2024, 24, 7417 10.1021/acs.nanolett.4c01604. 38836571 PMC11194845

[ref72] Alcón I.; Viñes F.; Moreira I. d. P.; Bromley S. T. Existence of multi-radical and closed-shell semiconducting states in post-graphene organic Dirac materials. Nat. Commun. 2017, 8 (1), 1957 10.1038/s41467-017-01977-4. 29208895 PMC5717056

[ref73] Ortiz R.; Catarina G.; Fernández-Rossier J. Theory of triangulene two-dimensional crystals. 2D Materials 2023, 10 (1), 015015 10.1088/2053-1583/aca4e2.

[ref74] Catarina G.; Henriques J. C. G.; Molina-Sánchez A.; Costa A. T.; Fernández-Rossier J. Broken-symmetry magnetic phases in two-dimensional triangulene crystals. Phys. Rev. Res. 2023, 5 (4), 43226 10.1103/PhysRevResearch.5.043226.

[ref75] Zhou Y.; Liu F. Realization of an antiferromagnetic superatomic graphene: Dirac Mott insulator and circular dichroism Hall effect. Nano Lett. 2021, 21 (1), 230–235. 10.1021/acs.nanolett.0c03579. 33263394

[ref76] Wu S.; Li M.; Phan H.; Wang D.; Herng T. S.; Ding J.; Lu Z.; Wu J. Toward two-dimensional π-conjugated covalent organic radical frameworks. Angew. Chem., Int. Ed. 2018, 57 (27), 8007–8011. 10.1002/anie.201801998. 29750390

[ref77] Henriques J.; Ferri-Cortés M.; Fernández-Rossier J. Designer spin models in tunable two-dimensional nanographene lattices. Nano Lett. 2024, 24 (11), 3355–3360. 10.1021/acs.nanolett.3c04915. 38427975 PMC10958603

[ref78] Wang W. L.; Yazyev O. V.; Meng S.; Kaxiras E. Topological frustration in graphene nanoflakes: magnetic order and spin logic devices. Phys. Rev.Lett. 2009, 102 (15), 157201 10.1103/PhysRevLett.102.157201. 19518670

[ref79] Mishra S.; Beyer D.; Eimre K.; Kezilebieke S.; Berger R.; Gröning O.; Pignedoli C. A.; Müllen K.; Liljeroth P.; Ruffieux P.; Feng X.; Fasel R. Topological frustration induces unconventional magnetism in a nanographene. Nature Nanotechnol. 2020, 15 (1), 22–28. 10.1038/s41565-019-0577-9. 31819244

[ref80] Lambson B.; Carlton D.; Bokor J. Exploring the Thermodynamic Limits of Computation in Integrated Systems: Magnetic Memory, Nanomagnetic Logic, and the Landauer Limit. Phys. Rev.Lett. 2011, 107 (1), 010604 10.1103/PhysRevLett.107.010604. 21797532

[ref81] Landauer R. Irreversibility and heat generation in the computing process. IBM J. Res. Dev. 1961, 5 (3), 183–191. 10.1147/rd.53.0183.

[ref82] Zuo L.; Ye L.; Li X.; Xu R.-X.; Yan Y.; Zheng X. Unraveling the Nature of Spin Coupling in a Metal-Free Diradical: Theoretical Distinction of Ferromagnetic and Antiferromagnetic Interactions. J. Phys. Chem. Lett. 2024, 15 (21), 5761–5769. 10.1021/acs.jpclett.4c01063. 38776132

[ref83] Henriques J.; Jacob D.; Molina-Sánchez A.; Catarina G.; Costa A. T.; Fernández-Rossier J. Beyond Spin Models in Orbitally Degenerate Open-Shell Nanographenes. Nano Lett. 2024, 24, 12928 10.1021/acs.nanolett.4c03416. 39374927 PMC11487622

[ref84] Catarina G.; Turco E.; Krane N.; Bommert M.; Ortega-Guerrero A.; Gröning O.; Ruffieux P.; Fasel R.; Pignedoli C. A. Conformational tuning of magnetic interactions in coupled nanographenes. Nano Lett. 2024, 24, 12536 10.1021/acs.nanolett.4c03518. 39348224

[ref85] Mermin N. D.; Wagner H. Absence of ferromagnetism or antiferromagnetism in one-or two-dimensional isotropic Heisenberg models. Phys. Rev. Lett. 1966, 17 (22), 1133 10.1103/PhysRevLett.17.1133.

[ref86] Gambardella P.; Dallmeyer A.; Maiti K.; Malagoli M. C.; Eberhardt W.; Kern K.; Carbone C. Ferromagnetism in one-dimensional monatomic metal chains. Nature 2002, 416 (6878), 301–304. 10.1038/416301a. 11907571

[ref87] Magda G. Z.; Jin X.; Hagymási I.; Vancsó P.; Osváth Z.; Nemes-Incze P.; Hwang C.; Biro L. P.; Tapasztó L. Room-temperature magnetic order on zigzag edges of narrow graphene nanoribbons. Nature 2014, 514 (7524), 608–611. 10.1038/nature13831. 25355361

[ref88] Jenkins S.; Rózsa L.; Atxitia U.; Evans R. F. L.; Novoselov K. S.; Santos E. J. G. Breaking through the Mermin-Wagner limit in 2D van der Waals magnets. Nat. Commun. 2022, 13 (1), 6917 10.1038/s41467-022-34389-0. 36376290 PMC9663506

[ref89] Maghrebi M. F.; Gong Z.-X.; Gorshkov A. V. Continuous symmetry breaking in 1d long-range interacting quantum systems. Phys. Rev. Lett. 2017, 119 (2), 023001 10.1103/PhysRevLett.119.023001. 28753374 PMC6467271

[ref90] Yu H.; Sun J.; Heine T. Predicting Magnetic Coupling and Spin-Polarization Energy in Triangulene Analogues. J. Chem. Theory Comput. 2023, 19 (12), 3486–3497. 10.1021/acs.jctc.3c00175. 37263582

[ref91] Sun Q.; Yang J.; Chan G. K.-L. A general second order complete active space self-consistent-field solver for large-scale systems. Chem. Phys. Lett. 2017, 683, 291–299. 10.1016/j.cplett.2017.03.004.

[ref92] White S. R. Density matrix formulation for quantum renormalization groups. Phys. Rev. Lett. 1992, 69 (19), 2863 10.1103/PhysRevLett.69.2863. 10046608

[ref93] Sharma P.; Truhlar D. G.; Gagliardi L. Magnetic coupling in a tris-hydroxo-bridged chromium dimer occurs through ligand mediated superexchange in conjunction with through-space coupling. J. Am. Chem. Soc. 2020, 142 (39), 16644–16650. 10.1021/jacs.0c06399. 32875795

[ref94] Olivares-Amaya R.; Hu W.; Nakatani N.; Sharma S.; Yang J.; Chan G. K. The ab-initio density matrix renormalization group in practice. J. Chem. Phys. 2015, 142 (3), 034102 10.1063/1.4905329. 25612684

[ref95] Cramer C. J.; Truhlar D. G. Density functional theory for transition metals and transition metal chemistry. Phys. Chem. Chem. Phys. 2009, 11 (46), 10757–10816. 10.1039/b907148b. 19924312

[ref96] Moreira I. d. P. R.; Illas F. A unified view of the theoretical description of magnetic coupling in molecular chemistry and solid state physics. Phys. Chem. Chem. Phys. 2006, 8 (14), 1645–1659. 10.1039/b515732c. 16633648

[ref97] Kowalczyk T.; Tsuchimochi T.; Chen P.-T.; Top L.; van Voorhis T. Excitation energies and Stokes shifts from a restricted open-shell Kohn-Sham approach. J. Chem. Phys. 2013, 138 (16), 164101 10.1063/1.4801790. 23635105

[ref98] Hait D.; Head-Gordon M. Highly accurate prediction of core spectra of molecules at density functional theory cost: Attaining sub-electronvolt error from a restricted open-shell Kohn–Sham approach. J. Phys. Chem. Lett. 2020, 11 (3), 775–786. 10.1021/acs.jpclett.9b03661. 31917579

[ref99] Hait D.; Zhu T.; McMahon D. P.; van Voorhis T. Prediction of excited-state energies and singlet–triplet gaps of charge-transfer states using a restricted open-shell Kohn–Sham approach. J. Chem. Theory Comput. 2016, 12 (7), 3353–3359. 10.1021/acs.jctc.6b00426. 27267803

[ref100] Shao Y.; Gan Z.; Epifanovsky E.; Gilbert A. T.; Wormit M.; Kussmann J.; Lange A. W.; Behn A.; Deng J.; Feng X.; Ghosh D.; Goldey M.; Horn P. R.; Jacobson L. D.; Kaliman I.; Khaliullin R. Z.; Kuś T.; Landau A.; Liu J.; Proynov E. I.; Rhee Y. M.; Richard R. M.; Rohrdanz M. A.; Steele R. P.; Sundstrom E. J.; Woodcock H. L. III; Zimmerman P. M.; Zuev D.; Albrecht B.; Alguire E.; Austin B.; Beran G. J. O.; Bernard Y. A.; Berquist E.; Brandhorst K.; Bravaya K. B.; Brown S. T.; Casanova D.; Chang C.-M.; Chen Y.; Chien S. H.; Closser K. D.; Crittenden D. L.; Diedenhofen M.; DiStasio R. A. Jr.; Do H.; Dutoi A. D.; Edgar R. G.; Fatehi S.; Fusti-Molnar L.; Ghysels A.; Golubeva-Zadorozhnaya A.; Gomes J.; Hanson-Heine M. W.; Harbach P. H.; Hauser A. W.; Hohenstein E. G.; Holden Z. C.; Jagau T.-C.; Ji H.; Kaduk B.; Khistyaev K.; Kim J.; Kim J.; King R. A.; Klunzinger P.; Kosenkov D.; Kowalczyk T.; Krauter C. M.; Lao K. U.; Laurent A. D.; Lawler K. V.; Levchenko S. V.; Lin C. Y.; Liu F.; Livshits E.; Lochan R. C.; Luenser A.; Manohar P.; Manzer S. F.; Mao S.-P.; Mardirossian N.; Marenich A. V.; Maurer S. A.; Mayhall N. J.; Neuscamman E.; Oana C. M.; Olivares-Amaya R.; O’Neill D. P.; Parkhill J. A.; Perrine T. M.; Peverati R.; Prociuk A.; Rehn D. R.; Rosta E.; Russ N. J.; Sharada S. M.; Sharma S.; Small D. W.; Sodt A.; Stein T.; Stück D.; Su Y.-C.; Thom A. J.; Tsuchimochi T.; Vanovschi V.; Vogt L.; Vydrov O.; Wang T.; Watson M. A.; Wenzel J.; White A.; Williams C. F.; Yang J.; Yeganeh S.; Yost S. R.; You Z.-Q.; Zhang I. Y.; Zhang X.; Zhao Y.; Brooks B. R.; Chan G. K.; Chipman D. M.; Cramer C. J.; Goddard W. A. III; Gordon M. S.; Hehre W. J.; Klamt A.; Schaefer H. F. III; Schmidt M. W.; Sherrill C. D.; Truhlar D. G.; Warshel A.; Xu X.; Aspuru-Guzik A.; Baer R.; Bell A. T.; Besley N. A.; Chai J.-D.; Dreuw A.; Dunietz B. D.; Furlani T. R.; Gwaltney S. R.; Hsu C.-P.; Jung Y.; Kong J.; Lambrecht D. S.; Liang W.; Ochsenfeld C.; Rassolov V. A.; Slipchenko L. V.; Subotnik J. E.; van Voorhis T.; Herbert J. M.; Krylov A. I.; Gill P. M.; Head-Gordon M. Advances in molecular quantum chemistry contained in the Q-Chem 4 program package. Mol. Phys. 2015, 113 (2), 184–215. 10.1080/00268976.2014.952696.

[ref101] Daugherty M. C.; Jacobse P. H.; Jiang J.; Jornet-Somoza J.; Dorit R.; Wang Z.; Lu J.; McCurdy R.; Tang W.; Rubio A.; Louie S. G.; Crommie M. F.; Fischer F. R. Regioselective On-Surface Synthesis of [3] Triangulene Graphene Nanoribbons. J. Am. Chem. Soc. 2024, 146, 15879 10.1021/jacs.4c02386. 38813680 PMC11177251

[ref102] Wen E. C. H.; Jacobse P. H.; Jiang J.; Wang Z.; McCurdy R. D.; Louie S. G.; Crommie M. F.; Fischer F. R. Magnetic interactions in substitutional core-doped graphene nanoribbons. J. Am. Chem. Soc. 2022, 144 (30), 13696–13703. 10.1021/jacs.2c04432. 35867847

[ref103] Zhao Y.; Jiang K.; Li C.; Liu Y.; Zhu G.; Pizzochero M.; Kaxiras E.; Guan D.; Li Y.; Zheng H.; et al. Quantum nanomagnets in on-surface metal-free porphyrin chains. Nat. Chem. 2023, 15 (1), 53–60. 10.1038/s41557-022-01061-5. 36280765

[ref104] Delgado A.; Dusold C.; Jiang J.; Cronin A.; Louie S. G.; Fischer F. R. Evidence for excitonic insulator ground state in triangulene Kagome lattice. arXiv preprint 2023, arXiv:2301.06171 10.48550/arXiv.2301.06171.

[ref105] Clair S.; de Oteyza D. G. Controlling a chemical coupling reaction on a surface: tools and strategies for on-surface synthesis. Chem. Rev. 2019, 119 (7), 4717–4776. 10.1021/acs.chemrev.8b00601. 30875199 PMC6477809

[ref106] Kolmer M.; Zuzak R.; Steiner A.-K.; Zajac L.; Engelund M.; Godlewski S.; Szymonski M.; Amsharov K. Fluorine-programmed nanozipping to tailored nanographenes on rutile TiO_2_ surfaces. Science 2019, 363 (6422), 57–60. 10.1126/science.aav4954. 30606840

[ref107] Kuc A.; Springer M. A.; Batra K.; Juarez-Mosqueda R.; Wöll C.; Heine T. Proximity Effect in Crystalline Framework Materials: Stacking-Induced Functionality in MOFs and COFs. Adv. Funct. Mater. 2020, 30 (41), 1908004 10.1002/adfm.201908004.

